# In Situ Raman Analysis of CO_2_—Assisted Drying of Fruit-Slices

**DOI:** 10.3390/foods6050037

**Published:** 2017-05-15

**Authors:** Andreas Siegfried Braeuer, Julian Jonathan Schuster, Medhanie Tesfay Gebrekidan, Leo Bahr, Filippo Michelino, Alessandro Zambon, Sara Spilimbergo

**Affiliations:** 1 Lehrstuhl für Technische Thermodynamik (LTT), Friedrich-Alexander-Universitaet Erlangen-Nuernberg (FAU), Am Weichselgarten 8, 91058 Erlangen, Germany; andreas.braeuer@fau.de (A.S.B.); julian.schuster@fau.de (J.J.S.); medhanie.gebrekidan@fau.de (M.T.G.); leo.bahr@fau.de (L.B.); 2 Erlangen Graduate School in Advanced Optical Technologies (SAOT), Friedrich-Alexander-Universitaet Erlangen-Nuernberg (FAU), Paul-Gordan-Straße 6, 91052 Erlangen, Germany; 3 Department of Industrial Engineering, University of Padova, via Marzolo 9, 35131 Padova, Italy; sara.spilimbergo@unipd.it (F.M.); alessandro.zambon@unipd.it (A.Z.)

**Keywords:** drying, fruit, carbon dioxide, high pressure, Raman spectroscopy, water, water content, in situ, pasteurization

## Abstract

This work explores the feasibility of applying in situ Raman spectroscopy for the online monitoring of the supercritical carbon dioxide (SC-CO_2_) drying of fruits. Specifically, we investigate two types of fruits: mango and persimmon. The drying experiments were carried out inside an optical accessible vessel at 10 MPa and 313 K. The Raman spectra reveal: (i) the reduction of the water from the fruit slice and (ii) the change of the fruit matrix structure during the drying process. Two different Raman excitation wavelengths were compared: 532 nm and 785 nm. With respect to the quality of the obtained spectra, the 532 nm excitation wavelength was superior due to a higher signal-to-noise ratio and due to a resonant excitation scheme of the carotenoid molecules. It was found that the absorption of CO_2_ into the fruit matrix enhances the extraction of water, which was expressed by the obtained drying kinetic curve.

## 1. Introduction

The removal of moisture from food products and in particular from fruits is one of the oldest preservation methods [1]. Indeed, decreased water activity inhibits the growth of microorganisms and therefore increases the shelf life of the product [2]. In order to reduce the drying time, conventional drying processes usually take place at elevated temperature [3]. However, treating fruits at elevated temperatures bears the risk that valuable and thermo-labile molecules, such as vitamins or carotenoids, might get degraded whereby the fruits will lose their nutrient and health value [4]. Also, the visual appearance of dried fruits can suffer from thermal treatment [5]. 

Supercritical carbon dioxide (SC-CO_2_) has been used as drying agent showing great potential as an alternative to low temperature drying techniques. Example applications are drying in the food industry [6,7,8,9], the production of organic and inorganic highly porous aerogels [10,11] and wood drying [12].

Conventional drying techniques themselves do not successfully inactivate microorganisms on the dried products, resulting in quick spoilage when the products are rehydrated and increasing the risk of foodborne illness [13]. Therefore, additional pasteurization treatments are often added after conventional drying. Unfortunately, conventional thermal pasteurization processes, like thermal drying processes, induce significant modifications of the qualitative and nutritional aspects of the fruits. This problem might be solved by the use of SC-CO_2_ as a drying agent. It has been largely demonstrated that SC-CO_2_ is a promising alternative to thermal pasteurization treatment. SC-CO_2_ drying is usually exploited at mild temperatures [14] and thus preserves the original properties of the fruits [15].

The SC-CO_2_ drying process combines the extraction of water from fruits and the reduction of the microbial load. While the former has been already investigated, the latter warrants further studies to be proven. Additionally, when the compressed CO_2_ is recirculated, this process also seems to feature manageable operational costs, which are superior to conventional drying [16].

In this context, we mainly investigated the possibility of using Raman spectroscopy for the in situ analysis of the SC-CO_2_ drying process, rather than analyzing the drying kinetics. The in situ Raman spectroscopy measurements during the SC-CO_2_ drying process were carried out in small, optically accessible lab-scale vessels. Based on these measurements, it might be possible to identify the optimal operational conditions, with respect to an economical operation of the process and with respect to the characteristics of the processed fruits, which is not the target of this study. Consequently, these optimal operational conditions could be translated to technological relevant process-scales in larger vessels. Additionally, Raman measurement could become a useful detection technique for the achievement/control of perfect drying conditions during large industrial processes. 

This manuscript focuses on the working principle of the measurement technique and on the evaluation of the in situ acquired spectra. The applicability of the Raman technique is demonstrated by analyzing two different example fruits: mango and persimmon. The applications of Fourier transform infrared (FTIR) absorption spectroscopy to supercritical drying, extraction and impregnation of thin layers are reviewed elsewhere [17].

## 2. Materials and Methods 

### 2.1. Preparation of Fruits and Computation of the Water Mass Ratio

Mango and persimmon were bought from the daily fruit and vegetable market in Padua, Italy. The fruits were cut into slices (2–5 mm thickness) before their insertion into the CO_2_-drier. They were dried inside the CO_2_-drier the same day of purchasing. During the CO_2_-drying experiments, the excitation laser beam was aligned through the cut interface of the fruit in order to record the Raman spectra effectively from the interior of the fruits and to avoid the possibility that the incident laser interferes with the peel of the fruits. 

The slices were weighed directly before the CO_2_-drying process ($m_{water}\left(t_{0}\right)+m_{dry\;Fruit}$), after the completion of the CO_2_-drying process ($m_{water}\left(t_{1}\right)+m_{dry\;Fruit}$) and after the removal of the residual moisture in a thermal dryer $m_{dry\;Fruit}$, when the free water contained in the fruit is finally equal to zero; $m_{water}\left(t_{2}\right)=0$. From these measurements, the mass ratio, expressed as:

(1) $$\varphi \left(t\right)=\frac{m_{water}\left(t\right)}{m_{dry\;Fruit}}\;.$$

This equation computes the mass of water and the mass of dry fruit tissue contained in the fruit slices.

### 2.2. Experimental CO_2_-Drying and Raman Setup

**Figure 1:**
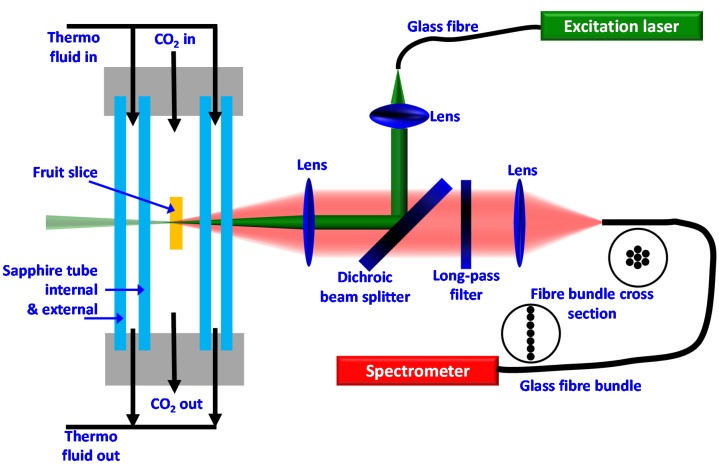
Schematic representation of the experimental setup comprising the double-tubed, optically accessible, high pressure sapphire CO_2_-drying vessel and the design of the Raman sensors.

Figure 1 shows a schematic representation of a Raman sensor together with the CO_2_-drying chamber. The fruit slice was positioned inside a double-tubed, optically accessible 50-mL high pressure sapphire vessel SC400, manufactured by Separex S.A.S (Champigneulles, France) and described in detail elsewhere [18]. The concentric double tube enables temperature conditioning of the vessel’s interior by purging a thermo-fluid (here water) through the ring-gap of the sapphire jacket, while still preserving optical accessibility. During the CO_2_-drying experiments, moderate operation conditions of 10 MPa, 313 K and a CO_2_ flow rate of 190 g min^−1^ were set. At these operation conditions, the bulk CO_2_ surrounding the fruit slice is in a supercritical state. The CO_2_ flow rate assures a gentle flow around the slices.

One Raman sensor was operated at an excitation wavelength of 532 nm, the other one was operated at 785 nm. In both Raman sensors the excitation light coming from the laser source is guided through a glass fibre, then collimated by a first convex lens, then guided via a dichroic mirror towards the fruit and focused via another convex lens into the tissue of the fruit. The dichroic mirror is highly reflective for the laser wavelength, but transparent for longer wavelengths. The Raman signals scattered from the focal spot (centre of probe volume) are imaged onto the glass fiber detection bundle using the same lens as a collimation lens, which already focused the excitation laser, and a second convex lens to focus the signals on the fiber. As the Raman signals are shifted to longer wavelengths with respect to the excitation wavelength, the Raman signals can pass the dichroic mirror. On the way to the detection fiber, the Raman signals are purified from the elastically scattered laser light, firstly by the dichroic mirror and secondly by the long-pass filter. The round-to-linear glass fiber bundle guides the Raman signals to the spectrometers (QE65000 Ocean Optics, one for the visible and one for the near-infrared spectral range), where they are dispersed and recorded as intensity-over-wavelength spectra and have to be converted and processed into intensity-over-Raman-shift spectra.

All lenses feature a diameter of 2′′ and a focal length of 100 mm. The lenses in the Raman sensor operated with 532 nm excitation wavelength are anti-reflection-coated for the visible spectral range. The lenses in the Raman sensor that we operated with an excitation wavelength of 785 nm are anti-reflection-coated for the red to near-infrared spectral range. Also, the glass fibers installed in the Raman sensors are optimized for the specific wavelengths.

The 785 nm excitation Raman sensor was operated with a power of less than 500 mW. For measurements with the 532 nm excitation Raman sensor, the laser power was reduced to a maximum of 250 mW, as higher laser powers mutated the tissue (photo-dissociation or photo bleaching) [19]. Approximately one fifth of the laser power reaches the measurement object. The remaining four fifths are deflected away when the laser beam passes the cylindrical double tube sapphire jacket of the drying-chamber. No degeneration of the tissue could be observed from the detected Raman signals and from visual inspection of the fruit tissue at the effective powers of 100 mW at 785 nm and 50 mW at 532 nm, respectively.

### 2.3. Evaluation of the Raman Spectra

The recorded spectra were background corrected and purified from fluorescence interference, according to the measures described in the results section. From the obtained pure Raman spectra, we extracted as a function of time t:

- the intensity $I_{water}\left(t\right)$ of the water stretch vibration band as the integral of the Raman spectra between 3110 and 3800 cm^−1^;
- the intensity $I_{C-H}$ of the carbon-hydrogen (C-H) vibration band of the fruit tissue as the integral of the Raman spectrum between 2800 and 3110 cm^−1^.

The water stretch vibration of water molecules is due to the permanent movement (vibration) of the two hydrogen nuclei towards and away from the oxygen nucleus. The C-H vibration in molecules containing carbon and hydrogen (biological molecules such as those in fruit tissue) is due to the permanent movement of the hydrogen nucleus towards and away from the carbon nucleus. Since $I_{water}\left(t\right)$ is proportional to the mass of water $m_{water}\left(t\right)$, and since $I_{C-H}$ is proportional to the mass of dry fruit tissue $m_{dry\;Fruit}\left(t\right)$, the water mass ratio at an instant of time in the fruit within the probed volume of the Raman sensor, expressed as:

(2) $$\varphi \left(t\right)=\frac{m_{water}\left(t\right)}{m_{dry\;Fruit}\left(t\right)}\propto \frac{I_{water}\left(t\right)}{\;I_{C-H}}\;,$$

can be set proportional to the ratio of evaluated Raman signal intensities. If the fruit tissue expands or shrinks during the CO_2_-drying process, the masses $m_{water}\left(t\right)$ and $\;m_{dry\;Fruit}$ within the probed volume change. The Raman signal intensities $I_{water}\left(t\right)$ and $\;I_{C-H}$ from the probed volume change accordingly and thus the computed water mass ratio φ(t) still shows the actual correct value. This is one of the advantages of considering the ratios of Raman signal intensities instead of absolute intensities.

## 3. Results and Discussion

**Figure 2:**
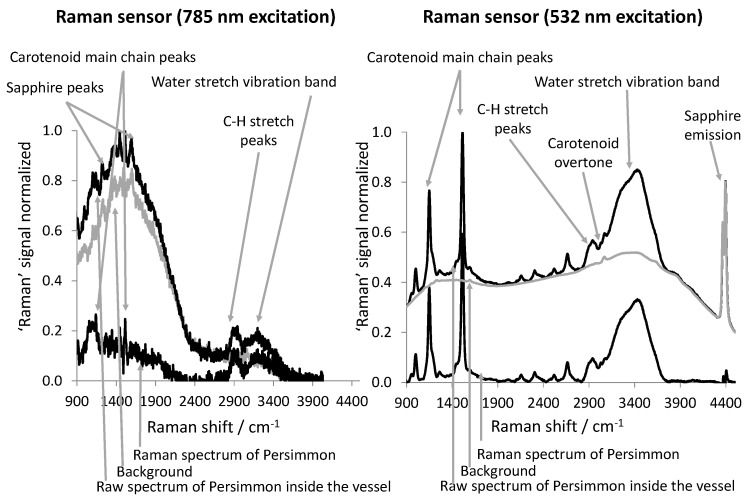
(**left**) Raw (black) spectrum and purified (black baseline) Raman spectrum from a piece of persimmon fruit inside the vessel acquired with the 785 nm excitation Raman sensor. (**right**) Raw (black) spectrum and purified (black baseline) Raman spectrum from a piece of persimmon fruit inside the vessel acquired with the 532 nm excitation Raman sensor.

Figure 2 shows spectra of a persimmon slice acquired with the 785 nm (left) and the 532 nm (right) excitation Raman sensors when the fruit slice was positioned inside the drying chamber before starting the SC-CO_2_ drying process.

The Raman spectrum excited with 532 nm is an average of two spectra, each recorded with an integration time of 10 s. The Raman spectrum excited with 785 nm is an average of five Raman spectra, each recorded with an integration time of 2 s. At longer integration times, the integrated signals (including the background) saturate the detector. The solid black spectra are normalized to one and represent the raw spectra. The grey solid spectra represent the background spectra that were subtracted from the raw spectra in order to obtain the pure Raman spectra. The pure Raman spectra are represented by the black solid spectra sitting on the “zero-baseline.” The spectra recorded with the two different Raman sensors are different with respect to their signal-to-noise ratios, the shape of their backgrounds and the shape of the Raman spectra themselves.

Though the Raman sensor operated at 532 nm utilized approximately half of the excitation power of the other Raman sensor, the signal-to-noise ratio of the spectra obtained is superior. Firstly, this is due the fact that the Raman scattering cross-sections scale approximately with the fourth power of the inverse excitation wavelength $\left(\lambda^{-4}\right)$, so that the 532 nm excitation Raman sensor generates approximately 2.4 times more Raman signal than the other sensor. Secondly, this is due the fact that the quantum efficiency of the back illuminated detectors of the spectrometers in the visible spectral range is approximately five times better than it is in the near-infrared spectral range. 

In the case of the 785 nm excitation Raman sensor, the background dominates the Raman spectrum at Raman shifts smaller than 2400 cm^−1^. This background emerges from light-matter interactions between the excitation laser radiation and the vessel (sapphire and cooling water between the sapphire tubes), and it disappears when the excitation laser is switched off. In the case of the 532 nm excitation Raman sensor, there are peaks observable in the spectrum, which could be attributed to the sapphire of the vessel. In both cases the raw spectrum was corrected with a background spectrum, which was acquired from the empty CO_2_-drying vessel with the respective laser switched on. This additional background correction method is suitable for the spectra obtained from the 785 nm excitation Raman sensor, while the spectra from the 532 nm excitation Raman sensor still show a broad fluorescence background, which originates from various fluorescing compounds contained in the fruit slice itself. Therefore, in this latter case, the remaining broadband fluorescence signal was fitted combining a Savitzky-Golay and a polynomic spline method, and then subtracted from the raw spectrum [20]. Therefore, the grey line labelled “Background” in the right part of Figure 2 is composed of the background detected from the empty vessel and the fluorescence originating from the fruit slice.

For Raman shifts larger than 2800 cm^−1^ in both cases, the pure Raman spectra feature a Raman band representing the carbon-hydrogen (C-H) stretch vibration and a broad Raman band representing the water stretch vibration. This band is broad compared to common Raman peaks, as the vibrational energy of water molecules become indistinct due to hydrogen bonds developing between water molecules and between water and other molecules that can potentially form hydrogen bonds [21,22,23]. Because of the fast decay of the quantum efficiency of the detector with an increase in wavelength, the water band once appears smaller (785 nm excitation Raman sensor) and once larger (532 nm excitation Raman sensor) than the C-H vibration. However, the main differences in the characteristics of the spectra appear at the spectral positions of the two carotenoid main chain peaks at $\sim 1150\;{\mathrm{cm}}^{-1}$ and $\sim 1500\;{\mathrm{cm}}^{-1}$. The Raman peak at $\sim 1150\;{\mathrm{cm}}^{-1}$ is due to the vibration between carbon nuclei that are linked via a single-bond (C-C vibration). The Raman peak at $\sim 1500\;{\mathrm{cm}}^{-1}$ is due to the vibration between carbon nuclei that are linked via a double bond (C = C vibration). The photo-physics and Raman characteristics of carotenoids have been previously reported [24,25,26,27,28,29,30,31]. The carotenoid peaks dominate the 532 nm excited Raman spectrum due to the resonant excitation [32,33], which also explains the visibility of the harmonic and combination modes between 2000 cm^−1^ and 2800 cm^−1^ [31]. The 785 nm excitation radiation does not have enough photon-energy for a near-resonant excitation. Therefore, the respective Raman transitions are not resonantly enhanced. A less intense enhancement explains the visibility of the two fundamental carotenoid peaks (C-C and C = C vibrations) in the Raman spectrum excited with 785 nm, according to the π-electron/phonon coupling mechanism as proposed by Castiglioni et al. [34]. This enhancement was later described by Parker et al. [31] in the context of carotenoids. The characteristic features of the Raman spectra that we described above for the persimmon sample are very similar as those for mango (data not shown). 

**Figure 3:**
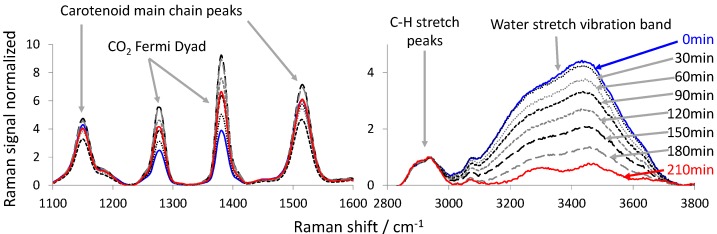
Temporal series of the processed Raman spectra acquired with the 532 nm excitation Raman sensor during an example drying experiment of a slice of persimmon.

**Figure 4:**
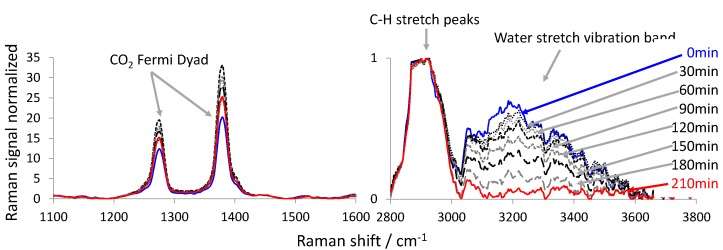
Temporal series of the processed Raman spectra acquired with the 785 nm excitation Raman sensor during an example drying experiment of a slice of persimmon.

Figure 3 and Figure 4 show the Raman spectra acquired during the CO_2_-drying of the persimmon using the 532 nm and the 785 nm excitation Raman sensors, respectively. In both figures, the spectral regions around the Raman Fermi dyad [35] of CO_2_ are shown between 1100 and 1600 cm^−1^ (left) and around the C-H stretch and the water stretch vibration between 2800 and 3800 cm^−1^ (right). All spectra are normalized to the maximum of the Raman signal of the C-H vibration between 2800 cm^−1^ and 3000 cm^−1^. Thus, the maximum of the Raman signal peak of the C-H vibration in Figure 3 and Figure 4 is equal to one. We chose this peak as a normalization standard, as it is clearly represented in all spectra, and we assumed that the number of C-H bindings within the fruit slice remains constant during the drying experiment. In Figure 3 and Figure 4 (right), it can be observed how the signal of the water stretch vibration band decreases with CO_2_-drying time, relative to the C-H peak. This indicates that the water is extracted out of the fruit slice and into the SC-CO_2_ flow that surrounds the sample. In Figure 3 and Figure 4 (left), the strong Fermi dyad of CO_2_ can be seen. These peaks are very dominant, as the drying vessel is flushed with SC-CO_2_. Therefore, there are many CO_2_ molecules present within the measurement volume of the Raman sensor, which also covers regions outside the fruit (depth of focus). Only in the case of the resonant Raman strategy (532 nm excitation wavelength) can the enhanced carotenoid peaks be observed next to the Fermi dyad. In the case of the 785 nm excitation, the carotenoid Raman peaks are simply eaten up in the noise of the spectra. In both cases the Raman signal of CO_2_ at 210 min is greater than at the beginning (0 min). Therefore, we assume that the CO_2_ diffuses into the fruit slice, while the water is extracted. This assumption is supported by the observation that we were able to foam persimmon slices when the CO_2_-drying vessel was depressurized quickly. In both cases a maximum CO_2_ Raman signal was found at a certain time between the beginning and the end of the drying experiment. This can be assigned to the solubility of CO_2_ inside the fruit-slice that changes as a function of the overall composition. A similar behaviour was observed by Quiño et al. [36] during drying of alcogels to aerogels using compressed CO_2_.

The carotenoid peaks show approximately the same intensity at the beginning and at the end of the drying experiment. During the drying, the intensity of the carotenoid peaks varied a little. Since we normalized the Raman spectra to the C-H Raman signal, an up-and-down of the carotenoid Raman signals can only be explained by the swelling or shrinkage of the matrix material of the fruit when CO_2_ penetrates and/or water exits the fruit [37].

**Figure 5:**
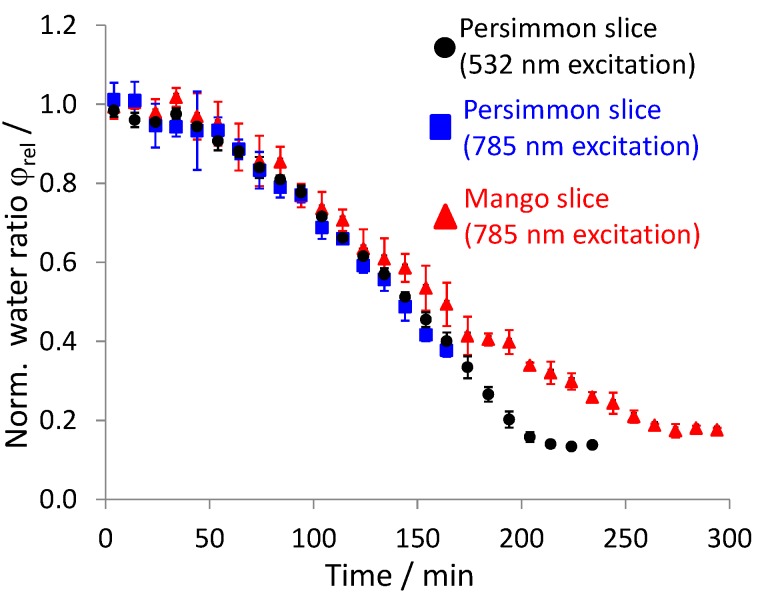
Drying kinetics of fruit slices measured with the Raman sensors. Mango slice of 2.73 g, persimmon slices of 2.09 g (785 nm excitation) and 3.48 g (532 nm excitation).

From the ratio of the corresponding Raman signal intensities, $\varphi \left(t\right)=\frac{m_{water}\left(t\right)}{m_{dry\;Fruit}\left(t\right)}\alpha \frac{I_{water}\left(t\right)}{\;I_{C-H}},$ the reduction of the water content in the fruit slice can be followed as a function of time, as is shown in Figure 5. The drying kinetics of the mango and the persimmon slice show that after a drying time of 250 and 200 min, respectively, a nearly-stationary value is reached. Apparently, the removal of this last portion of water with CO_2_-drying would take much longer. We therefore stopped the CO_2_-drying experiments of the slices [38]. During one drying experiment (persimmon slice with 785 nm excitation), we ran out of CO_2_ after 170 min and thus had to stop before the stationary value was reached. Nevertheless, the drying curves for the two persimmon fruits are nearly identical (within the range of the error bars) during the first 170 min, irrespectively of which excitation wavelength, 532 nm or 785 nm, we used in the Raman sensor.

The determination of the precision of the Raman measurement techniques is challenging, as the multiple repetition of drying experiments at exactly the same conditions is impossible. This is due to the varying nature of the biological samples. There are no fruit slices that feature exactly the same properties. Therefore, the precision derived from the mutual repetition of drying experiments with different fruit slices from the same fruit would always be corrupted by the varying properties of the fruit slices. Consequently, we computed the precision that is shown in the form of the error bars in Figure 5, according to the strategy that follows. During the drying experiment, we made two Raman measurements per minute. Related to the entire drying time of 200 min and longer (see Figure 5), the temporal sampling period of 30 s is negligibly short. In other words, there should be nearly no detectable drying progress from one Raman measurement to the other. The scatter we observe from one Raman measurement to the other then is the margin of error of the Raman technique. Therefore, the entire drying kinetics curves, which originally were resolved in temporal increments of 30 s, are shown as mean drying curves in Figure 5. Each data point is the average of five subsequently recorded Raman measurements. For clear presentation, we show one mean data point for every two minutes. The error bars given in Figure 5 are the standard deviations of the single Raman measurements from the mean drying curve.

The right-hand sections of Figure 3 and Figure 4 show that the Raman signal intensity of the water stretching vibration (normalized to the C-H vibration Raman signal peak maximum) is higher in the case of the 532 nm excitation laser. This explains why in Figure 5 the error bars of the data points of the drying curve measured with the 532 nm excitation Raman sensor are smaller than the error bars of the data points of the drying curves measured with the 785 nm excitation Raman sensor. 

The reliability of the obtained measurement results is confirmed by the circumstance that very similar drying kinetics can be obtained for the same fruit (persimmon), irrespective of the excitation wavelength used for the Raman sensor.

Furthermore, the drying curves in Figure 5 show an interesting behavior. One would have expected a steep negative slope at the beginning, which flattens towards the end [39]. However, in Figure 5, the drying curves start flat, become steeper and subsequently flatten again. The spectra in Figure 3 and Figure 4 show that the content of CO_2_ relative to the C-H vibration changes over time. During the drying time, the CO_2_ content first increases, reaches a maximum and then decreases. Therefore, we conclude that the mass transport of water out of the fruit slice is also a function of the CO_2_ content. The more CO_2_ that is contained inside the fruit, the easier the water can be removed from the fruit, since the CO_2_ swells the fruit. In the experiments carried out without convection (data not shown), the fruit was positioned inside a quiescent supercritical CO_2_ environment at 10 MPa and 313 K. Thus, we were neither able to monitor an enrichment of the CO_2_ content inside the fruit nor a detectable reduction of the water mass ratio. Because of this evidence, we conclude that the CO_2_ cannot penetrate into the fruit before some water has been removed. Otherwise, we would have observed an enrichment of CO_2_ in the experiment without convection. Eventually, some of the water has to be removed from the fruit before the CO_2_ can penetrate into the fruit, which then enhances the extraction of the remaining water. Thus, the drying curve first is flat and then becomes steeper. At late drying times, the concentration gradient of water still remaining inside the fruit slice and in the bulk CO_2_-rich phase grows smaller, which decelerates the extraction of water and again flattens the drying curve towards the end.

Table 1 summarizes the masses and the normalized water mass ratio $\varphi_{rel}$ (normalized to the water mass ratio before drying) of the fruit slices before CO_2_-drying ($m_{water}\left(t_{0}\right)+m_{dry\;Fruit}$), after CO_2_-drying ($m_{water}\left(t_{1}\right)+m_{dry\;Fruit}$), and after complete drying in a thermal dryer $m_{dry\;Fruit}$.

The normalized water mass ratio, in which 100% corresponds to the original fruit slice before drying and 0% corresponds to the completely dried fruit slice after the thermal drying in the oven, was computed from the masses of the fruit slices. As shown in Table 1, using this weighing method, the normalized water mass ratios after CO_2_ drying for the two persimmon slices have the values of $\varphi_{rel}=0.077$ (persimmon slice 1) and $\varphi_{rel}=0.124$ (persimmon slice 2), which are approximately half as much as those measured with the in situ Raman method (compare with the end values in Figure 5). Therefore, we conclude that a substantial amount of water is spilled out of the fruit slice together with the CO_2_ when the pressure is released from the drying vessel at the end of the CO_2_ drying process.

## 4. Conclusions

We dried fruit slices in an optically accessible high pressure vessel, which we purged continuously with supercritical carbon dioxide (CO_2_). We analysed in situ the drying kinetics of the drying process without taking probes out of the vessel, by applying a remote Raman measurement technique. For Raman excitation wavelengths, we chose 532 nm and 785 nm. Both Raman sensors could be utilized for the in situ analysis of the water mass content of the fruit slices, as both sensors reveal one Raman signal attributable to the “dry” fruit material, in this case, the C-H stretch Raman signal, and another attributable to the water inside the fruit, in other words, the water stretch vibration. Due to the wavelength dependency of the Raman scattering cross-section and the wavelength-dependent quantum efficiency of the detectors, the signal-to-noise ratio of the spectra acquired with the 532 nm excitation Raman sensor is significantly superior to the one recorded with the 785 nm excitation Raman sensor. Nevertheless, both Raman sensors revealed the drying curves of the fruit slices, which are characterized by initially slow kinetics, then fast kinetics and again slow kinetics towards the end of the CO_2_-drying process. Furthermore, the 532 nm excitation Raman sensor resonantly excites and thus enhances the Raman signals of the carotenoids, which are not detectable with the 785 nm excitation Raman sensor. Valuable information can be extracted from the dominant carotenoid peaks concerning the characteristics of the dried fruits. The central wavenumber of the second carotenoid peak at approximately 1500 cm^−1^ is known to be a function of the lengths of the conjugated carbon-carbon main chain [24] and of the local environment [40]. Therefore, if the drying-temperature or the laser power damages or degrades the carotenoid molecules, a shift in the central peak position can be observed. Furthermore, the monitoring of the intensity of the carotenoid peaks makes possible the analysis of whether some of the carotenoids are detracted from the fruit together with the water.

In this study, the drying experiments have been carried out in a double-walled sapphire vessel, which significantly attenuated the laser beam and the Raman signals and which also showed an interference on the Raman spectra. For this reason, further studies are in progress to obtain similar results with significantly less laser excitation power, using a high pressure vessel equipped with plain windows.

## Figures and Tables

**Table 1:** Masses and normalized water mass ratios of the fruit slices before the CO_2_ drying experiment, after CO_2_ drying and after complete drying in a thermal dryer (normalized to the water mass ratio before drying).

		Persimmon Slice 1	Persimmon Slice 2	Mango Slice
Before drying	($m_{water}\left(t_{0}\right)+m_{dry\;Fruit}$)/g	3.48	2.09	2.73
	Norm. water ratio $\varphi_{rel}$	1	1	1
After CO_2_ drying	($m_{water}\left(t_{1}\right)+m_{dry\;Fruit}$)/g	0.74	0.61	0.44
	Norm. water ratio $\varphi_{rel}$	0.077	0.124	0.026
After thermal drying	$m_{dry\;Fruit}$/g	0.51	0.40	0.38
	Norm. water ratio $\varphi_{rel}$	0	0	0
